# Role of Serotonin, Membrane Transporter, and 5-HT2 Receptors in Pathogenesis of Atherosclerotic Plaque Formation in Immature Heterozygous Low-Density Lipoprotein-Receptor-Deficient Mice

**DOI:** 10.3390/ijms26136184

**Published:** 2025-06-26

**Authors:** Dinara Sadykova, Razina Nigmatullina, Karina Salakhova, Evgeniia Slastnikova, Liliya Galimova, Chulpan Khaliullina, Elena Gafurova, Dmitry Tsyplakov

**Affiliations:** 1 Department of Hospital Pediatrics, Kazan State Medical University, 420012 Kazan, Russia; karina.salakh@mail.ru (K.S.); e.slastnikova@mail.ru (E.S.); lilu1@inbox.ru (L.G.); chulpandanilevna@yandex.ru (C.K.); 2 Department of Normal Physiology, Kazan State Medical University, 420012 Kazan, Russia; razinar@mail.ru; 3 Children’s Republican Clinical Hospital, 420138 Kazan, Russia; 4 Forensic Histology Department, Republican Bureau of Forensic Medical Examination, 420029 Kazan, Russia; 5 Department of General Pathology, Kazan State Medical University, 420012 Kazan, Russia

**Keywords:** serotonin, membrane serotonin transporter, cardiovascular disease, familial hypercholesterolemia, serotonin receptors, vascular smooth muscle cells, mice

## Abstract

Familial hypercholesterolemia leads to the early development of cardiovascular diseases at a young age due to the prolonged exposure of the arterial vessel wall to high concentrations of atherogenic lipids. Serotonin plays a significant role in the development and progression of atherosclerotic processes. Monoamine has a damaging effect on the vascular wall, stimulates the proliferation of vascular smooth muscle cells and fibroblasts, and participates in platelet activation and aggregation. The aim of the work was the demonstration of the importance of serotonin, transporters, and receptors in the pathogenesis of atherosclerotic plaque formation. The study was performed on immature mice of the C57BL/6JGpt-Ldlr^em1Cd82^/Gpt (*Ldlr*^+/−^) line (main group) and C57BL/6 mice of comparable age and sex demographics (control group). Morphological manifestations of early signs of atherosclerosis (pre-lipid stage and lipoidosis stage, which were confirmed by Sudan III staining) in the gene-modified mice’s aorta were determined. Morphological changes in the aorta correlated with changes in the left ventricle of the heart, where lipid content also increased. No atherosclerotic changes in the control-group mice were detected. A statistically significant increase in the expression of the membrane serotonin transporter and 5HT2A and 5HT2B receptors in both the aorta and left ventricle was also found in the animals of the main group. Serotonin and its receptors and transporter may become new therapeutic targets for the treatment and prevention of atherosclerotic vascular lesion progression in children and adults.

## 1. Introduction

Cardiovascular disease (CVD) has been the leading cause of death and disability in the world’s population for the past decades. According to the Global Burden of Disease (GBD) study, conducted in 2021, about 19 million people will die from circulatory diseases, and the DALY index (disability-adjusted life years lost) is 438 million life years lost [1].

It has currently been established that dyslipidaemia is the one of the main causes of cardiovascular disease development and progression [2]. The prolonged exposure of high concentrations of atherogenic lipoproteins to the arterial vessel wall leads to the premature development of CVD of atherosclerotic genesis [3].

The animal models of atherosclerosis play important roles in studying the molecular and pathophysiological mechanisms underlying atherosclerotic vascular changes [4]. The first evidence that atherosclerosis can occur in laboratory animals was presented by Ignatowski in 1908. Atherosclerotic lesions were revealed in the aortic walls of rabbits fed with food enriched with animal protein (mainly meat, milk, and egg yolk). Since then, various animal species such as rabbits, mice, rats, guinea pigs, hamsters, birds, and dogs have been used for experiments [5]. Mice constitute the most widely used animal models in experimental studies of atherosclerosis nowadays. The ease of breeding, low cost of maintenance, possibility of genetic manipulations, and relatively short period of atherosclerosis development make rodent models ideal models for studying atherosclerotic vascular changes [4,6]. Two classical transgenic mice models’ creation on the background of the C57BL/6 line was a ‘breakthrough’ in the history of atherosclerosis research in the late 20th century: apoprotein E-deficient (Apoe^−/−^) and low-density lipoprotein receptor (LDLR)-deficient (Ldlr^−/−^) [6,7]. LDLR is a membrane receptor, located on the surfaces of many cell types, that mediates the endocytosis of cholesterol-rich LDL and thus maintains plasma low-density lipoprotein (LDL-cholesterol) levels [4,8].

Familial hypercholesterolemia (FH) is a monogenic disease characterized by high levels of total cholesterol (TC) and LDL-cholesterol in blood plasma from birth, which subsequently leads to early atherosclerotic lesions of the main vessels and the development of cardiovascular disease at a young age [9]. Today, there are approximately 35 million people with familial hypercholesterolaemia worldwide, including 6.8–8.5 million children [10]. The development of FH is caused by abnormalities in the LDL receptor gene in 95% of cases [11].

The heterozygous form of FH is usually asymptomatic during the first decades of life and, consequently, there are no clinical manifestations of the disease. However, despite the asymptomatic course, patients with heterozygous FH are exposed to lifelong exposure to extremely high FH; these patients are the group at high or very high cardiovascular risk [12].

Myocardial infarction, stroke, and peripheral arterial disease are the culminations of the atherosclerotic process. The pathological process begins with the accumulation of abnormal lipids in the intima of vessels, which corresponds to the ‘reversible’ stage. Then the changes progress to a late stage, in which the core of extracellular lipids is covered by a fibrotic-muscular capsule, and everything ends with thrombosis, vessel rupture, or acute ischaemia [13]. It is known that most of the clinical manifestations of CVD occur in adulthood, which led to the erroneous belief that this pathology is absent in children. However, studies in recent years have shown that atherosclerosis is a multistage process that begins in utero and progresses throughout life [3,14]. This is fundamentally important, because it radically changes our idea of the ‘stages’ of the process, and justifies the need to detect and correct dyslipidaemia already in early childhood.

Recent studies have shown that serotonin plays a significant role in the initiation and progression of CVD. Serotonin, 5-hydroxytryptamine (5-HT), is a neurotransmitter, a monoamine that was first described as a vasoconstrictor [15]. In the body, 5-HT fulfils a wide range of functions and is divided into central and peripheral types depending on the site of expression [16]. In the central nervous system (CNS), it controls such brain functions as autonomic neuron activity, stress response, body temperature, sleep, mood, and appetite [16,17]. However, all these processes are regulated by only about 5% of the body’s own 5-HT, which is produced in the brainstem suture nuclei [18]. The remaining 95% of 5-HT is synthesized in the periphery, mainly by enterochromaffin cells in the gut involving the enzyme tryptophan hydroxylase 1 (TPH1) [18,19]. Serotonin practically does not pass through the intact blood–brain barrier [20]. After synthesis in the enterochromaffin cells of the intestine, serotonin is released into blood plasma, where it is absorbed by platelets with the help of the membrane serotonin transporter (SERT) [15]. SERT is an intracellular protein that is expressed on the platelet membrane surface and belongs to the family of Na^+^/Cl^−^-dependent solute transporters (SLC6) [21]. Once in the platelet cytoplasm, 5-HT is sequestered by the vesicular monoamine transporter (VMAT) into intracellular dense granules or cleaved by monoamine oxidase (MAO) [21,22]. 5-HT is metabolized in the liver and eventually excreted from the body as the main metabolite, 5-hydroxyindoleacetic acid (5-HIAA) [23].

In peripheral tissues, 5-HT influences the regulation of the vascular tone, intestinal peristalsis, heart rate, respiratory activity, haemostasis, immune response, and embryonic development [15,16]. Elevated 5-HT concentrations have been described in arterial hypertension, carotid atherosclerosis, and coronary heart disease [15]. A number of studies have shown that serotonin plays a key role in the development and progression of cardiovascular diseases of atherosclerotic genesis [24]. 5-HT, acting on the vessel walls, increases platelet activation and aggregation, contributing to the development of thrombosis. Serotonin promotes vasoconstriction, mitogenesis, vascular smooth muscle cell (VSMC) proliferation, and macrophage foam cell formation [25]. Serotonin can also act indirectly by enhancing the release and activity of other vasoconstrictors such as angiotensin and norepinephrine [26].

The existence of a large number of functions of 5-HT is due to the diversity of its receptors [16]. Receptors 5-HT1, 5-HT2, 5-HT4, and 5-HT7 are responsible for the peripheral effects of serotonin on the cardiovascular system [19]. Receptors of the 5-HT2 subfamily were first described in 1979 and are subdivided into subtypes 5-HT2A, 5-HT2B, and 5-HT2C [18,27]. Many cell types in peripheral tissues express 5-HT2A and 5-HT2B receptors, including platelets, fibroblasts, lymphocytes, and myocytes, VSMCs [28]. Expression of the 5-HT2C receptor has not been detected in the body periphery [18]. 5-HT2 receptors belong to the G-protein-coupled receptor (GPCR) superfamily [29]. Cholesterol participates in GPCR activation by two mechanisms: direct (changes in the receptor structure due to direct interaction with cholesterol) and indirect (changes (deformation) in the membrane structure) [30].

Today, the issue of the relationship between antidepressants, namely drugs that affect serotonin—selective serotonin reuptake inhibitors (SSRIs) and atherosclerotic cardiovascular diseases—remains controversial. The currently available literature data is contradictory and does not provide a clear answer. In a case–control study by Sauer et al., patients taking SSRIs were found to have a lower risk of myocardial infarction than those taking tricyclic or atypical antidepressants [31]. The proposed mechanism is the inhibition of platelet activation, and the long-term use of SSRIs depletes platelet serotonin stores, which, in the case of myocardial infarction, may reduce the amount of monoamine released and reduce the area of myocardial damage [18]. In a study of more than 2000 people, SSRI use was not associated with a reduced risk of cardiovascular disease [32].

There are also opposite data in the literature. In an experimental study in female primates, chronic SSRI use increased the extent of atherosclerosis in the right common carotid artery by an average of 60% [33]. This was consistent with data from a meta-analysis that aimed to determine whether there was an association between depression and carotid intima-media thickness (cIMT)—a marker of early atherosclerosis. The results showed that cIMT was significantly higher in patients with depression compared to controls [34].

SSRIs, by increasing the concentration of free serotonin in the blood plasma, can lead to the increased activation of 5-HT2B receptors on fibroblasts and cardiomyocytes, which will lead to the development of fibrosis and myocardial hypertrophy. As a result, the pumping function of the heart decreases [18]. At the same time, high concentrations of serotonin in the blood can provoke the development of serotonin syndrome, which manifests itself, among other things, as tachycardia, hypertension, and the disruption of other autonomic functions, mental processes, and the regulation of neuromuscular transmission [35]. Serotonin receptors are present in VSMCs and fibroblasts of the vascular wall, and serotonin, the concentration of which increases in the blood plasma due to the use of SSRIs, can participate in the remodeling of blood vessels, increasing their rigidity, causing hypertrophic growth of the muscle layer.

Despite the fact that serotonin was discovered more than 70 years ago, the currently available literature data do not fully explain the pathogenetic mechanisms of serotonin influence on the development of cardiovascular pathology, including atherosclerotic vascular lesions. Familial hypercholesterolemia constitutes an optimal model for studying the pathogenetic mechanisms of serotonin involvement in the atherosclerotic process.

Mice with a knockout of the *Ldlr*^−/−^ gene most closely mimic the lipid abnormalities and atherosclerotic processes observed in humans [36]. The absence of functional LDLR receptor is found in people with familial hypercholesterolemia, which makes the low-density lipoprotein receptor deficiency model the most useful and convenient for studying hereditary dyslipidaemia [5]. In this work, a study was conducted on immature *Ldlr*^+/−^ mice, which corresponded to the heterozygous form of familial hypercholestrinemia. The heterozygous form of familial hypercholestrinemia is one of the most common genetically determined lipid metabolism disorders and occurs in one in three-hundred-and-thirteen people [9,10].

The aim of the study was to demonstrate the importance of serotonin, the membrane transporter, and its receptors in the pathogenesis of atherosclerotic plaque formation. The data obtained will contribute to a better understanding of the mechanism of development and progression of atherosclerotic vascular alteration at both molecular and pathophysiological levels.

## 2. Results

The study was conducted on 20 mice aged 5–7 weeks: 10 mice of C57BL/6JGpt-Ldlr^em1Cd82^/Gpt (*Ldlr*^+/−^)—the main group and 10 mice of C57BL/6—the control group.

### 2.1. Control Group

**Figure 1:**
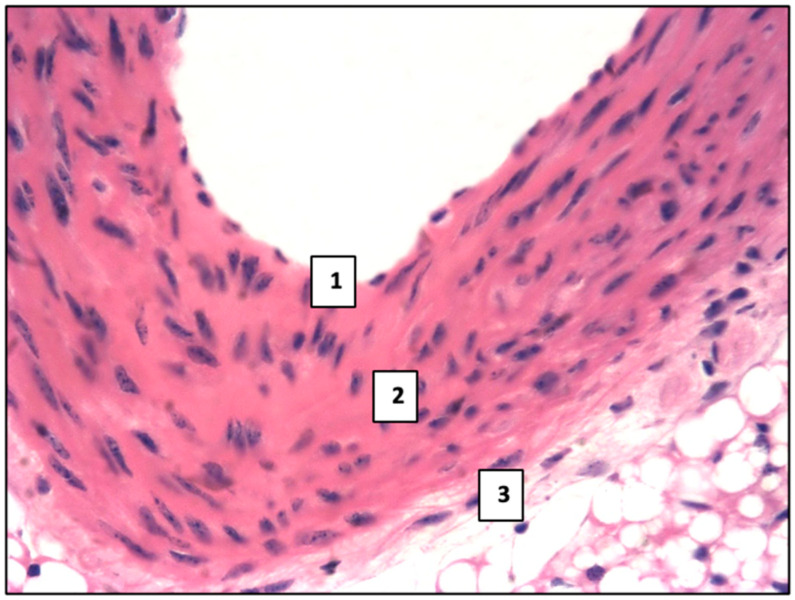
Normal histological structure of the aorta in mice: 1—inner sheath, 2—middle sheath, and 3—adventitial sheath. Control group. Haematoxylin and eosin staining. ×400.

In the aorta, the inner sheath (intima), middle sheath, and outer (adventitial) sheath were clearly defined (Figure 1). The intima was represented by a single layer of squamous endothelial cells with a weakly expressed subendothelial layer of loose connective tissue. The middle sheath contained elastic fibers and smooth muscle cells. The outer sheath was constructed of loose fibrous connective tissue with the presence of thick elastic and collagen fibers. Adipose tissue, judging from Sudan III staining, was detected only in the adventitial sheath.

**Figure 2:**
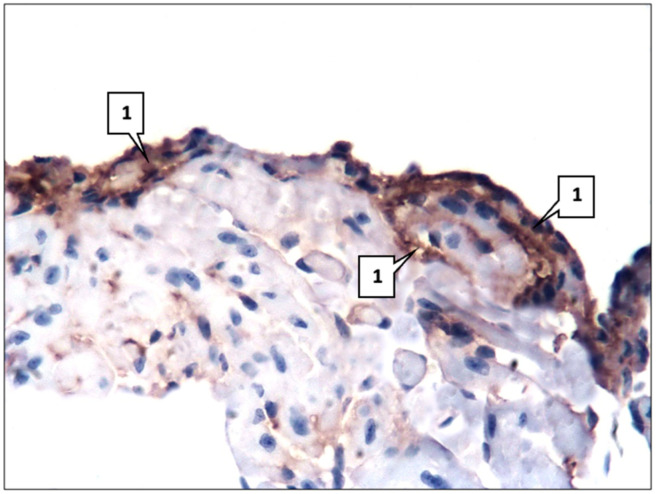
mAb expression against SERT in the mouse aorta (1). Control group. LSAB method with hematoxylin staining. ×400.

Immunohistochemical (IHC) analysis showed positive expressions of all used monoclonal antibodies (mAbs). Thus, the numbers of SERT-, 5HT2A-, and 5HT2B-positive cells were 16.2 ± 1.18%, 18.9 ± 1.22%, and 21.5 ± 1.46%, respectively. At the same time, mAbs against SERT and 5HT2A were expressed predominantly in endothelium and smooth muscle cells (Figure 2), and mAbs against 5HT2B were expressed in fibroblasts as well.

**Figure 3:**
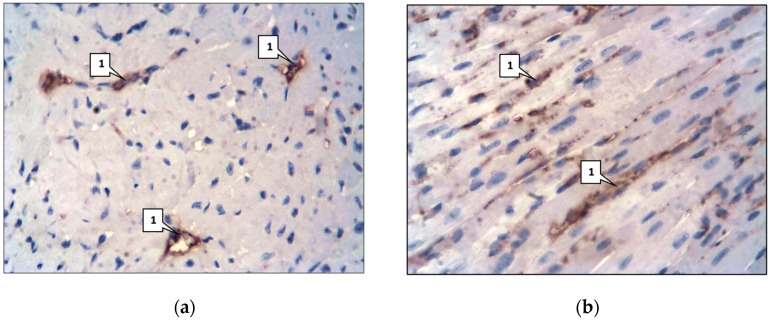
mAb expression against SERT. Control group. LSAB method with hematoxylin staining. ×400: expression (**a**) in the endothelium in mouse myocardial vessels (1); (**b**) in mouse cardiomyocytes (1).

The left ventricle of the heart, as well as the aorta, had a normal histological structure. The endocardium, myocardium, and epicardium were clearly defined in its wall. The endocardium was represented by a single layer of endotheliocytes and a thin subendothelial layer of connective tissue. The myocardium was formed by muscle fibers from cardiomyocytes. It also contained fibroblasts, blood vessels, and collagen fibers. The epicardium consisted of a thin connective tissue layer covered with mesothelium. Fatty tissue was detected predominantly in the epicardium whereas in the endocardium and myocardium, the lipid content was insignificant—only 0.09 ± 0.02% of the total area of the left ventricular slice. Immunohistochemically, as well as in the aorta, the expression of mAbs against SERT, 5HT2A, and 5HT2B was detected. A distinctive feature was a higher content of SERT- and 5HT2A-positive cells as they were also expressed in cardiomyocytes—27.4 ± 2.13% and 39.9 ± 2.78%, respectively. At the same time, SERT was detected predominantly in the vascular endothelium and cardiomyocytes (Figure 3).

### 2.2. Main Group

**Figure 4:**
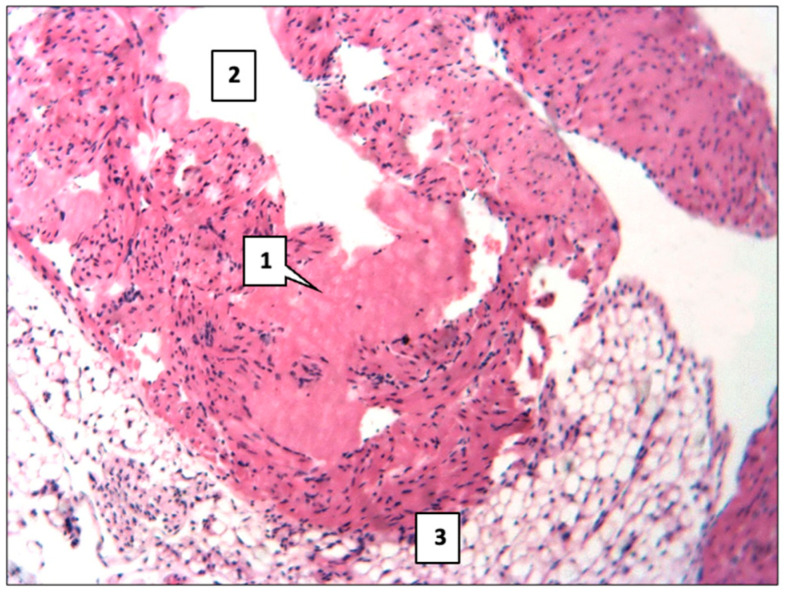
Deposition of eosinophilic hyaline-like masses (1) in the aortic intima of mice. 2—vessel lumen; 3—adventitial sheath. Main group. Haematoxylin and eosin staining. ×200.

**Figure 5:**
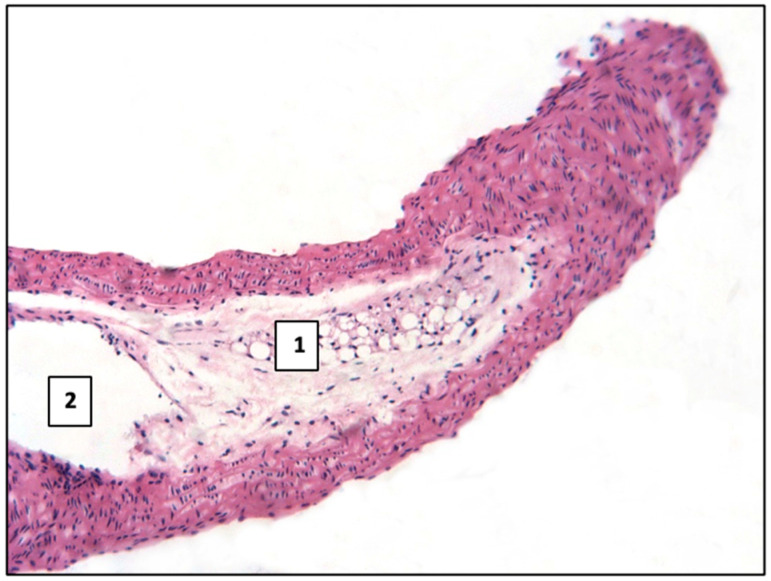
Lipid accumulation in the form of focal infiltration with the onset of atherosclerotic plaque formation (1) bulging into the lumen (2) of the aorta in mice. Main group. Haematoxylin and eosin. ×200.

In the majority of observations in the aorta, there was focal endothelial damage, increased permeability in the form of plasma saturation, the accumulation of plasma proteins, and fibrinogen in the inner membrane. Mucoid swelling, the destruction of separate elastic and collagen fibers, and the proliferation of smooth muscle cells were noted. This morphological picture corresponds to the earliest (pre-lipid) stage of atherosclerosis. In some cases, more pronounced changes were observed in the aorta. Thus, eosinophilic hyaline-like masses were deposited in the intima (Figure 4) or lipid accumulation in the form of focal infiltration with the beginning of atherosclerotic plaque formation, bulging into the aortic lumen (Figure 5).

**Figure 6:**
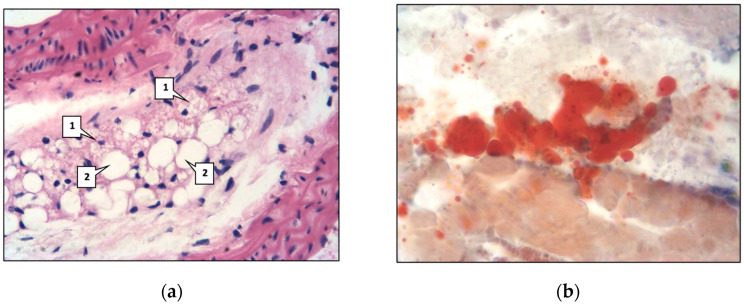
Mouse aorta. Main group: (**a**) Xanthoma cells (1); lipids (2). ×400. (**b**) Lipids in the aortic intima of mice. Sudan III staining. ×400.

Lipids were detected in individual vascular smooth muscle cells and macrophages, which are called ‘foamy’ or ‘xanthoma’ cells (Figure 6a). Lipids were detected by Sudan III staining (Figure 6b); the area occupied by them in the aortic intima and middle aortic sheath was 0.82 ± 0.09%. Such a morphological picture corresponds to the second microscopic stage of atherosclerosis, defined as lipoidosis.

**Figure 7:**
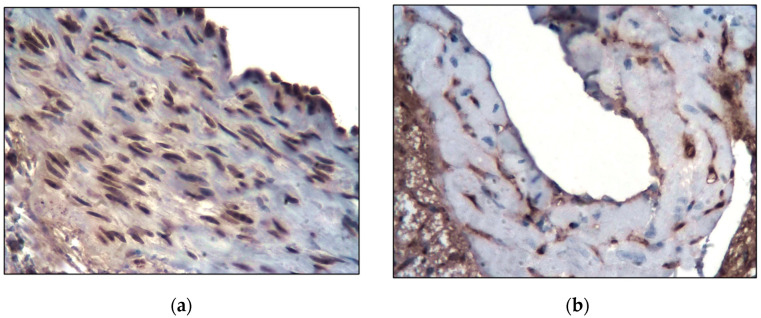
mAb expression against 5HT2A and 5HT2B. Main group. Mouse aorta. LSAB method with hematoxylin staining. ×400: (**a**) mAb expression against 5HT2A; (**b**) mAb expression against 5HT2B.

Immunohistochemical analysis showed a significant increase in the expression of mAbs against SERT, 5HT2A, and 5HT2B (Figure 7) in the aorta compared with the control group (Table 1).

**Figure 8:**
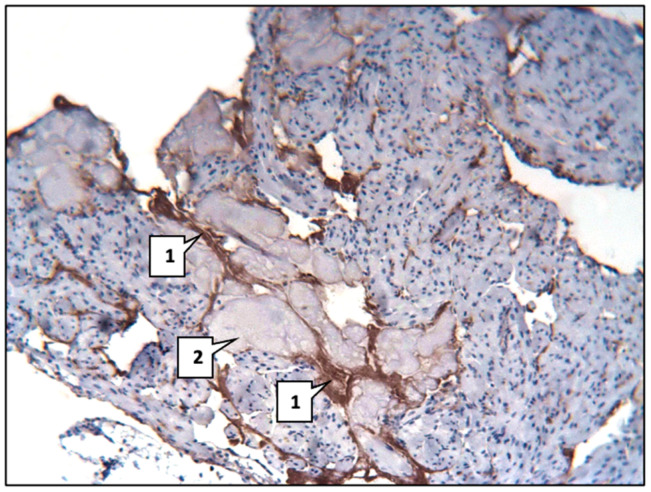
Expressed reaction of mAbs vs. SERT (1) in the area of hyaline-like mass deposition (2). Main group. LSAB method with haematoxylin staining. ×200.

The most pronounced reaction of mAbs, especially against SERT, was observed in the area of eosinophilic hyaline-like masses and lipid deposition (Figure 8).

**Figure 9:**
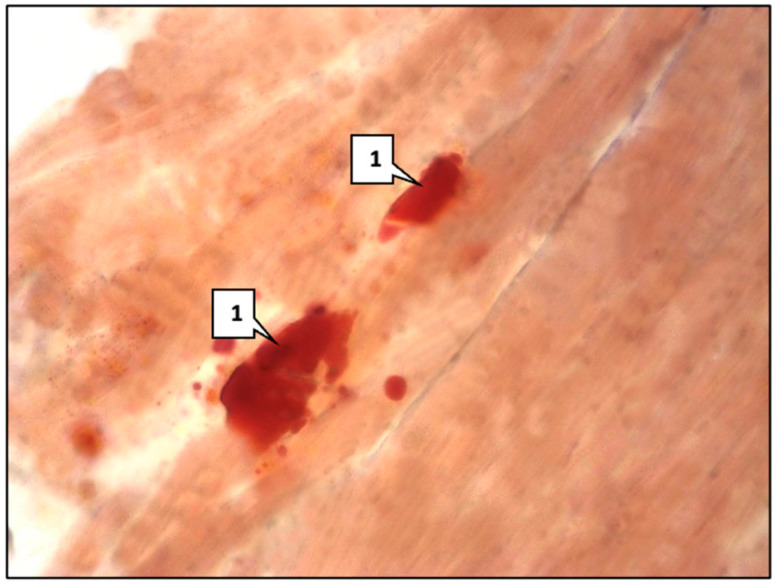
Lipids (1) in the myocardial stroma of mice. Main group. Sudan III staining. ×400.

**Figure 10:**
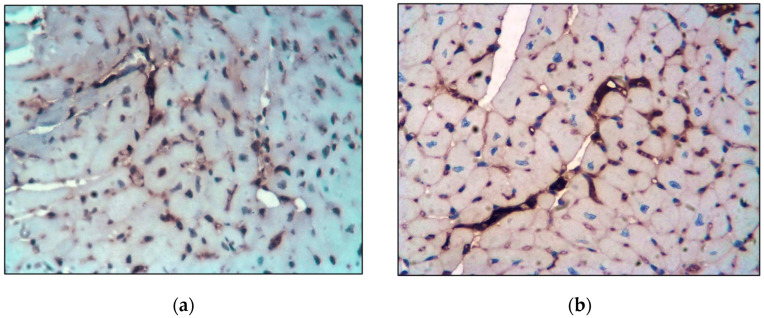
mAb expression against 5HT2A and 5HT2B. Main group. Myocardium. LSAB method with hematoxylin staining. ×400: (**a**) mAb expression against 5HT2A; (**b**) mAb expression against 5HT2B.

In the left ventricle of the heart, in parallel with the aorta, certain changes occurred. Thus, the lipid area increased in the endocardium and myocardium to 1.7 ± 0.011% (Figure 9) (Table 2), and the expression of mAbs against SERT, 5HT2A, and 5HT2B increased compared with control-group animals (Figure 10) (Table 3).

## 3. Discussion

Our study showed the presence of morphological manifestations of early signs of atherosclerosis in genetically modified mice, which was confirmed by histological methods of investigation. In the majority of cases, there was a predominantly pre-lipid stage of atherosclerosis and only in single cases was there a lipoidosis stage, which was confirmed by Sudan III staining. None of the observations showed the presence of later stages of atherosclerosis—liposclerosis, atheromatosis, ulceration, and atherocalcinosis. Morphological changes in the aorta correlated with changes in the left ventricle of the heart, where lipid content was also increased. Also, in animals of the main group, a statistically significant increase in the expression of mAbs against SERT, 5HT2A, and 5HT2B was detected.

Experimental studies have shown that animals with a mutation of the *Ldlr* gene most closely mimic human disorders of lipid metabolism and atherosclerotic processes [36]. Arai et al., in their study, found that atherosclerotic lesions in the aortic root were determined in sexually mature heterozygous mice with low-density lipoprotein receptor deficiency [37]. Similar data were obtained with heterozygous hamsters with *Ldlr* deficiency [38].

Atherosclerosis is a chronic disease that progresses throughout life and begins already in childhood [39]. It has been found that the degree of intima surface coverage by atherosclerotic lesions is associated with increased concentrations of TC, LDL, and triglycerides, as well as with a lower concentration of high-density lipoproteins (HDLs) [40]. FH is characterized by increased levels of LDL in plasma, which leads to their excessive accumulation in the arterial intima [39,41]. Atherogenic lipids are modified by enzymes and oxidized into proinflammatory particles that provoke an innate inflammatory response within the intima. Fat droplets accumulate in the cytoplasm of smooth muscle cells [39]. These changes are considered as early manifestations of human atherosclerotic disease and are characterized by the appearance of lipid-containing spots and streaks, so-called fatty streaks, in the intima of arteries [42]. Lipid spots appear in arteries from early childhood. At the age of 10 years, lipid spots occupy about 10% of the aortic intima, and by the age of 25 years, 30% to 50%. Lipoidosis occurs from 10–15 years of age in coronary arteries [43]. The present study showed that experimental animals with *Ldlr*^+/−^ gene mutation, whose age according to the age periodization of mice corresponded to 5–17 years, showed initial morphological signs of the atherosclerotic process—the pre-lipid stage and lipoidosis stage. The area of lipids in the endocardium and myocardium of the left ventricle of the heart in genetically modified mice was significantly higher compared with that of the animals of the control group. In C57BL/6 mice, lipids in the aortic intima and middle aortic sheath were not determined. The results of these kinds of studies are practically not found in the specialized scientific literature as the main part of such work is devoted to the study of atherosclerosis at later stages.

Serotonin is an intermediate product of tryptophan metabolism, most of which is synthesized by enterochromaffin cells in the intestine. Recent studies have shown that the sites of peripheral 5-HT biosynthesis are not limited to the gastrointestinal tract [19]. Cardiomyocytes; neuroendothelial cells of the lung, kidney, and adrenal gland; pancreatic B cells; and adipocytes are also capable of producing serotonin [19,44]. It has been proven that there is a local 5-hydroxytryptaminergic system capable of serotonin synthesis in peripheral arteries [44].

In our study, we found that mice with impaired cholesterol metabolism and developing atherosclerosis have an increased expression of mAbs against SERT, 5HT2A, and 5HT2B compared to control-group animals, which was consistent with other in vitro and in vivo studies [45]. In the periphery, serotonin is involved in the regulation of many cardiovascular functions: in addition to controlling blood pressure and heart rate, the monoamine is also ‘actively involved’ in the development and progression of atherosclerotic processes through its two classical functions—platelet aggregation and VSMC proliferation [46,47]. 5-HT2 serotonin receptors are involved in this process [46]. Serotonin release from platelets initiates the migration and proliferation of VSMCs into the intima, which leads to the thickening of vessel walls. VSMCs are the predominant cell type in atherosclerotic plaques [48]. In the lesion focus, VSMCs maintain the stability of atheromatous plaques by forming a fibrous capsule and producing extracellular matrix. However, in the early stage of atherosclerosis, VSMCs ‘play the opposite role’. The extracellular matrix produced at the initial stages of plaque formation can bind to apolipoproteins, which aggravates lipid accumulation in the subintima [49]. Unlike skeletal muscle cells, which are finally differentiated, mature VSMCs are highly plastic and capable of phenotypic changes in response to changes in environmental signals [50,51]. Under physiological conditions, VSMCs exhibit limited proliferative, migratory, and synthetic activity. In this state, their main phenotype is contractile, which is necessary for the regulation of vasomotor tone in blood vessels [48]. The cascade of reactions occurring in hypercholesterolemia leads to the switch of VSMCs to the synthetic phenotype, which is accompanied by the loss of contractile markers, transition to diamond-shaped morphology, and a marked increase in proliferation, migration, and protein synthesis [50]. Synthetic VSMCs secrete chemokines and cytokines, constituting the microenvironment of the inflammatory plaque, which further activates and promotes the phenotypic transition of VSMCs to macrophage-like (increased inflammatory reactions and release of matrix metalloproteinases), myofibroblast-like (the reorganization of the extracellular matrix, collagen deposition), and osteoblast-chondrocyte-like (vascular calcification) states [48]. VSMCs also play an important role in the formation of foam cells in the arterial plaque. Allahverdian et al. showed that about 50% of the total number of foam cells in the intima of human coronary arteries are derived from VSMCs rather than monocytes [52]. As early as in a 1986 study, it was proved that bovine aortic VSMCs in vitro responded to serotonin by increasing VSMC DNA synthesis. This effect was attenuated by the addition of serotonin receptor antagonists [53]. Watada et al. demonstrated that the daily administration of sarpogrelate (5-HT2A and 5-HT2B receptor antagonist) for 8 weeks reduced aortic intima thickening in rats [54]. The use of sarpogrelate in rabbits reduced the area of atherosclerotic plaques and slowed the progression of atherosclerosis due to an antiproliferative effect on smooth muscle cells [55].

The present study showed that the expression of mAbs against 5HT2A were higher in mice with atherosclerosis than in control animals. The 5-HT2A receptor was first discovered in the rat brain [28]. Later, its expression was confirmed in various brain regions, as well as in human smooth muscle cells, fibroblasts, cardiomyocytes, and platelets [18]. The activation of the 5-HT2A receptor causes various physiological effects in peripheral tissues. Besides participating in the proliferation and differentiation of VSMCs, the 5-HT2A receptor activates platelet aggregation, increases cardiac muscle contractility, and increases blood pressure [28]. The pharmacological inhibition of 5-HT2A receptor activity has cardioprotective effects on the heart [18]. Ketanserin (5-HT2A receptor antagonist) improves haemodynamic and neurohumoral changes in patients with heart failure [56]. In an experimental study by French scientists, the increased expression of 5-HT2A receptor in mice with cardiac hypertrophy was detected using immunohistochemistry [45].

The 5-HT2B receptor is expressed in cardiovascular tissues including myocardial, endothelial, and smooth muscle cells and fibroblasts [27]. The distributions of the 5-HT2B receptor protein in rodent and human tissues are similar, as are their pharmacologies, which facilitates the extrapolation of physiological and pharmacological results of 5-HT2B receptor studies from rodents to humans [57]. The 5-HT2B receptor is involved in many cardiovascular diseases, including cardiomyopathy, valvular heart disease, and pulmonary arterial hypertension [27]. Cardiovascular expression and the activation of the receptor can lead to proliferation of myofibroblasts [58]. The 5-HT2B receptor is a critical regulator of cardiac development. Its ablation in mouse embryos has resulted in embryonic and neonatal death due to cardiac defects; at the same time, increased 5-HT2B expression in mouse hearts has caused hypertrophic cardiomyopathy and excessive mitochondrial proliferation [18,59]. 5-HT2B receptors activate fibroblasts, which leads to connective tissue overgrowth and increased vascular stiffness [24]. The increased expression of 5-HT2B receptor in both cardiac fibroblasts and activated myofibroblasts promotes excessive scar formation, leading to adverse remodeling and impaired cardiac function after myocardial infarction [60]. Fong et al. determined that hyperlipidaemic Apoe^−/−^ mice had higher expressions of the 5-HT2B receptor in the aortic root than control animals with normal lipid levels [61]. This was consistent with our results. The expression of 5HT2B mAbs in heterozygous mice was higher in both the aorta and left ventricle.

As in the case of the 5-HT2B receptor in our study, mice in the main group showed an increased expression of mAbs against SERT compared to the control group. SERT is a bidirectional transporter that regulates intracellular and extracellular serotonin levels [62]. Cardiomyocytes, VSMCs, and vascular endothelial cells express the membrane transporter [20]. In previous studies, it was shown that mice with SERT deficiency developed cardiac fibrosis and heart valve damage [63]. Depending on the level of serotonin in blood plasma, the functional characteristics of the transporter change. An increase in plasma serotonin concentration leads to an increase in plasma membrane SERT levels and enhanced serotonin uptake. As 5-HT levels continue to increase, serotonin transporter performance decreases below threshold values. The high level of 5-HT in plasma limits its own uptake by platelets by suppressing SERT and changing the functional characteristics of the transporter [21,64]. SERT conformational changes are also dependent on cholesterol concentration as it is also able to interact with SERT, altering its configuration and function [65]. This lipid increases the affinity of the membrane transporter for serotonin [66]. A decrease in cholesterol level leads to a decrease in the activity of the membrane carrier through a change in its conformation, which decreases the affinity of SERT for serotonin and, consequently, the rate of its transport [67].

## 4. Materials and Methods

### 4.1. Animals

The study was conducted on C57BL/6JGpt-Ldlr^em1Cd82^/Gpt (*Ldlr*^+/−^) mice aged 5–7 weeks (main group) and C57BL/6 mice of the corresponding age and sex (control group). According to the age periodization of mice [68], the age of the mice was comparable to that of children from 5 to 17 years of age.

To obtain an experimental group of mice of the C57BL/6JGpt-Ldlr^em1Cd82^/Gpt (*Ldlr*^+/−^) line–mice of C57BL/6JGpt-Ldlr^em1Cd82^/Gpt (*Ldlr*^−/−^) line (cat. no. T001464; GemPharmatech Co., Ltd., Nanjing, China) were crossed with mice of the C57BL/6 line (‘Stolbovaya’ laboratory animal nursery of FSBI SCBT FMBA of Russia, Moscow, Russia). C57BL/6 mice from the control group were also purchased from the laboratory animal nursery ‘Stolbovaya’ of FSBI SCBT FMBA of Russia.

Animals were housed at room temperature with a 12/12 h light/dark cycle, indoor relative humidity ranging from 45% to 65%. Extraneous sound signals were minimized. Animals were housed in standard plastic cages of 3–4 individuals each. The housing conditions for all mice were the same. Standard feed for mice in the control group, high-fat feed for the main group, and water were available ad libitum before the start of the study.

For morphological and immunohistochemical studies, organ fragments (left ventricle, aorta) were taken from mice of two groups. Material for research in mice was collected postmortem after euthanasia of each animal (use of injectable drugs in a dosage exceeding the therapeutic dosage in accordance with interstate standard (GOST) 33215-2014) [69].

The study was approved by the Research Ethics Committee of Kazan State Medical University (protocol #5 dated 21 May 2024). All experiments were conducted in accordance with the ethical principles and regulations recommended by the European Science Foundation (ESF) and the Helsinki Declaration on Humane Treatment of Animals.

### 4.2. Morphological and Immunohistochemical Studies

The aorta and left ventricle of the heart were studied. Part of the material was fixed in 10% neutral formalin according to Lilly or Bowen’s fluid. According to the generally accepted method [70], after the appropriate running in alcohols of increasing concentration, treatment in xylene and casting in paraffin followed. Paraffin sections 4–5 µm thick were made on a Leica SM 2000R microtome (Leica Microsystems, Wetzlar, Germany). The obtained preparations were stained with haematoxylin and eosin. The other part was used for making cryostat sections, which were stained with Sudan III. For immunohistochemical study [71], we used a set of monoclonal antibodies (mAbs), the characteristics of which are presented in Table 4.

mAb binding to cellular elements was determined using the standard biotin–streptavidin–peroxidase method (DAKO: LSAB^®^ + System-HRP, code K0690) (Dako, Glostrup, Denmark) with diaminobenzidine as a chromogen. Preparations were additionally stained with Mayer’s gamatoxylin and encapsulated in Canadian balsam or DAKO special media (Ultramount, Faramount, code S302580-2) (Dako, Glostrup, Denmark).

Microscopic examination was performed using an ‘Axioscop-Zeiss AG’ microscope (Zeiss, Jena, Germany).

Lipid area was determined on histological sections stained with Sudan III using a random step morphometric grid from S.B. Stefanov [72]. For counting cells with different immunohistochemical phenotypes, we used the morphometric ocular grid of G.G. Avtandilov [73].

For a quantitative study of the cellular composition, a morphometric ocular grid was used, which was fixed inside the microscope eyepiece. It contained 25 points that were visualized in the field of view when viewing the microscope slide. The objects were studied at high magnification: eyepiece ×10, objective ×90 (immersion). The number of points corresponding to different cellular elements was counted. The field of view on the microscope slide was changed 40 times, randomly moving it on the microscope stage, repeating the count each time. In this way, 1000 points were taken into account, falling on different cells. The total number of points (1000) was taken as 100%. The percentage of different cell types was then calculated.

### 4.3. Statistical Analysis

Statistical processing of data was performed using STATISICA 8.0 program. Normal distribution of a sign was stated at *p* > 0.05 (Shapiro–Wilk test). Nonparametric methods of statistical analysis were used otherwise. In case of normal distribution of a sign, the arithmetic mean (M) and standard deviation (SD) were calculated. Reliability of differences between groups was calculated using Student’s t-criterion.

## 5. Conclusions

The presented data support our assumption that serotonin, the membrane serotonin transporter, and 5-HT2 receptors are involved in the pathogenesis of cardiovascular diseases of atherosclerotic genesis. Our study revealed initial morphological manifestations of early signs of atherosclerosis and increased expressions of mAbs against SERT, 5HT2A, and 5HT2B in heterozygous low-density lipoprotein-receptor-deficient mice. Serotonin and its receptors and transporter may become novel therapeutic targets for the treatment and prevention of progression of atherosclerotic vascular lesions in children and adults.

## Figures and Tables

**Table 1:** Contents of IHC-positive cellular elements in the intima and middle aortic sheath (in % of the total number of cells, Mean ± SD).

	Control Group	Main Group	*p* ^1^
SERT + cells	16.2 ± 1.18	25.3 ± 1.97	<0.05
5HT2A + cells	18.9 ± 1.22	24.9 ± 1.80	<0.05
5HT2B + cells	21.5 ± 1.46	32.4 ± 2.05	<0.05

^1^ *p*—the level of statistical significance of differences.

**Table 2:** Lipid area in the aortic intima and middle aortic sheath, as well as in the endocardium and myocardium of the left ventricle of the heart (in % of the total slice area, Mean ± SD).

	Control Group	Main Group	*p* ^1^
aorta	-	0.82 ± 0.09	
heart	0.09 ± 0.02	1.7 ± 0.011	<0.05

^1^ *p*—the level of statistical significance of differences.

**Table 3:** Contents of IHC-positive cell elements in endocardium and myocardium of the left ventricle of the heart myocardium of the left ventricle of the heart (in % of the total number of cells, Mean ± SD).

	Control Group	Main Group	*p* ^1^
SERT + cells	27.4 ± 2.13	36.3 ± 2.25	<0.05
5HT2A + cells	39.9 ± 2.78	48.3 ± 2.99	<0.05
5HT2B + cells	19.5 ± 1.74	28.6 ± 2.05	<0.05

^1^ *p*—the level of statistical significance of differences.

**Table 4:** Characteristics of the used monoclonal antibodies.

Antigen	Clone	Secondary Antibodies	Working Breeding	Manufacturing Company
SERT	polyclonal, code P31645	Goat Anti-Rabbit IgG (H+L) HRP	1:200	Affinity Biosciences (Cincinnati, OH, USA)
5HT2A	polyclonal, code P28223	Goat Anti-Rabbit IgG (H+L) HRP	1:250	Affinity Biosciences (Cincinnati, OH, USA)
5HT2B	polyclonal, code P41595	Goat Anti-Rabbit IgG (H+L) HRP	1:200	Affinity Biosciences (Cincinnati, OH, USA)

## Data Availability

All data are contained within this article.
